# Multimodal imaging documentation of rapid evolution of retinal changes in handheld laser-induced maculopathy

**DOI:** 10.1186/s40942-015-0014-7

**Published:** 2015-09-01

**Authors:** Elona Dhrami-Gavazi, Winston Lee, Chandrakumar Balaratnasingam, Larisa Kayserman, Lawrence A. Yannuzzi, K. Bailey Freund

**Affiliations:** 1 Vitreous-Retina-Macula Consultants of New York, 460 Park Avenue, 5th Floor, New York, NY 10022 USA; 2 The LuEsther T. Mertz Retinal Research Center, Manhattan Eye Ear and Throat Hospital, New York, NY USA; 3 Department of Ophthalmology, Edward S. Harkness Eye Institute, College of Physicians and Surgeons, Columbia University, New York, NY USA; 4 Department of Physiology and Pharmacology, Centre for Ophthalmology and Visual Sciences, Lions Eye Institute, University of Western Australia, Perth, Australia; 5 Retina Consultants of New Jersey, New Jersey, NJ USA

**Keywords:** Handheld laser-induced maculopathy, Multimodal imaging

## Abstract

**Purpose:**

To use multimodal imaging to document the relatively rapid clinical evolution of handheld laser-induced maculopathy (HLIM). To demonstrate that inadvertent ocular injury can result from devices mislabeled with respect to their power specifications.

**Methods:**

The clinical course of a 17-year-old male who sustained self-inflicted, central macular damage from a 20–25 s direct stare at a red-spectrum, handheld laser pointer ordered from an internet retailer is provided. Retrospective review of multimodal imaging that includes fundus photography, fluorescein angiography, MultiColor reflectance, eye-tracked spectral domain optical coherence tomography (SD-OCT), fundus autofluorescence, and microperimetry is used to describe the evolving clinical manifestations of HLIM in the first 3 months.

**Results:**

Curvilinear bands of dense hyperreflectivity extending from the outer retina and following the Henle fibers were seen on SD-OCT immediately after injury. This characteristic appearance had largely resolved by 2 weeks. There was significant non-uniformity in the morphological characteristics of HLIM lesions between autofluorescence and reflectance images. The pattern of lesion evolution was also significantly different between imaging modalities. Analysis of the laser device showed its wavelength to be correctly listed, but the power was found to be 102.5–105 mW, as opposed to the <5 mW described on the label.

**Conclusion:**

While the immediate SD-OCT characteristics are highly specific for handheld laser -induced maculopathy, this finding can undergo rapid resolution in the span of several days. In the absence of this finding, other multimodal imaging clues and a careful history may aid in recognizing this diagnosis. A greater awareness regarding inaccurate labeling on some of these devices could help reduce the frequency of this preventable entity.

## Background

Since the first report by Zamir et al. [1], a great number of additional cases of handheld laser-induced maculopathy (HLIM) have appeared in the ophthalmic and general medical literature. A recent increase in the frequency of these reports may be related to an increase in the availability of laser pointers intended for the public. These devices appear to be easily obtained through loosely regulated internet-based retailers. Various case reports and case series have characterized the clinical manifestations of this entity and have described the patterns that distinguish self-inflicted from accidental occurrences [1–8]. Self-inflicted cases of HLIM may manifest characteristic linear streaks on color photographs and other imaging modalities which will typically show a vertical pattern in the superior macula. This finding may relate to a discomfort-induced Bell’s phenomenon when staring at an intense light. Conversely, the accidental and peer-inflicted cases of HLIM typically appear as rounder lesions in the central macula [3]. Adolescent males are the typical demographic to present with HLIM and yellow/grey-colored lesions are commonly identified in the central and superior macula in the immediate period following injury [3]. Characteristic curvilinear hyperreflective bands are also seen on spectral domain optical coherence tomography (SD-OCT) imaging during this period [2–4].

The temporal course of HLIM immediately following injury, has not been documented in depth, but chronological studies that have reported mid- and long-term findings have suggested that the clinical manifestations of this disease evolve at a rapid rate. It is therefore important to understand the temporal characteristics of laser-induced maculopathy in order to distinguish it from other entities that may present with similar clinical signs. We present a patient with HLIM whose disease course in the 3 months after injury was closely and frequently documented with multimodal imaging. This report provides new information about the natural course and multimodal imaging characteristics of HLIM.

## Case presentation

A 17-year-old male was referred for evaluation of a 4 day history of decreased central vision in his right eye. He was under treatment for attention deficit hyperactivity disorder with 5 mg per day dexmethylphenidate hydrochloride extended release but was otherwise healthy. Past ocular history and family ocular history were both unremarkable. During the evaluation, the patient was pointedly asked whether he had been exposed to a hand held laser pointer. Reluctantly, he admitted that his visual symptoms occurred immediately after staring directly at the light emitted from a red laser pointer for 20–25 s. His father had purchased this device over the internet.

**Fig. 1:**
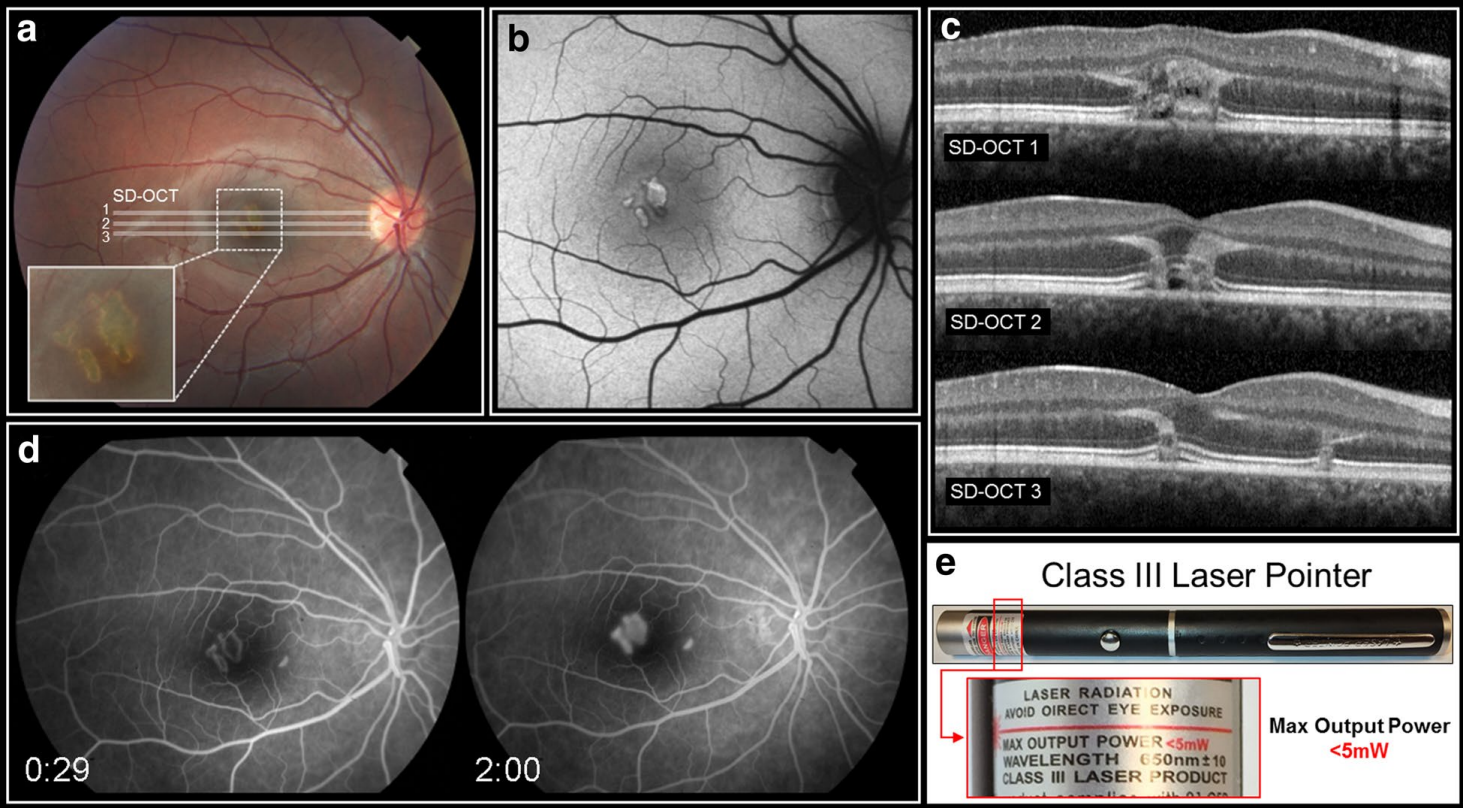
Funduscopic findings at presentation as documented via multimodal imaging. **a** Color photograph of the right eye taken 1 day after the injury shows sharply demarcated, “excavated” green-grey lesions with yellow borders situated in a vertical-oblique fashion in the central macula. **b** Fundus autofluorescence taken 1 day after the injury shows central hyperfluorescence of the lesions with a thin surrounding rim of hyperautofluorescence. **c** Spectral domain optical coherence tomography images of three different sections taken 1 day after the injury show curvilinear bands of dense hyperreflectivity that extend from the interdigitation layer and ellipsoid zone of the photoreceptors upwards, ending at the level of the outer plexiform layer. The hyperreflective lesions appear to follow the Henle fibers. There are small hyporeflective cavities beneath the fovea. **d** Fluorescein angiography taken 1 day after the injury shows hyperfluorescence of the lesions with minimal leakage. An additional satellite lesion, not detected with color photos and autofluorescence, is evident nasal to the fovea. **e** Photograph of the device used in the injury with a magnified view (*inset*) of its label

**Fig. 2:**
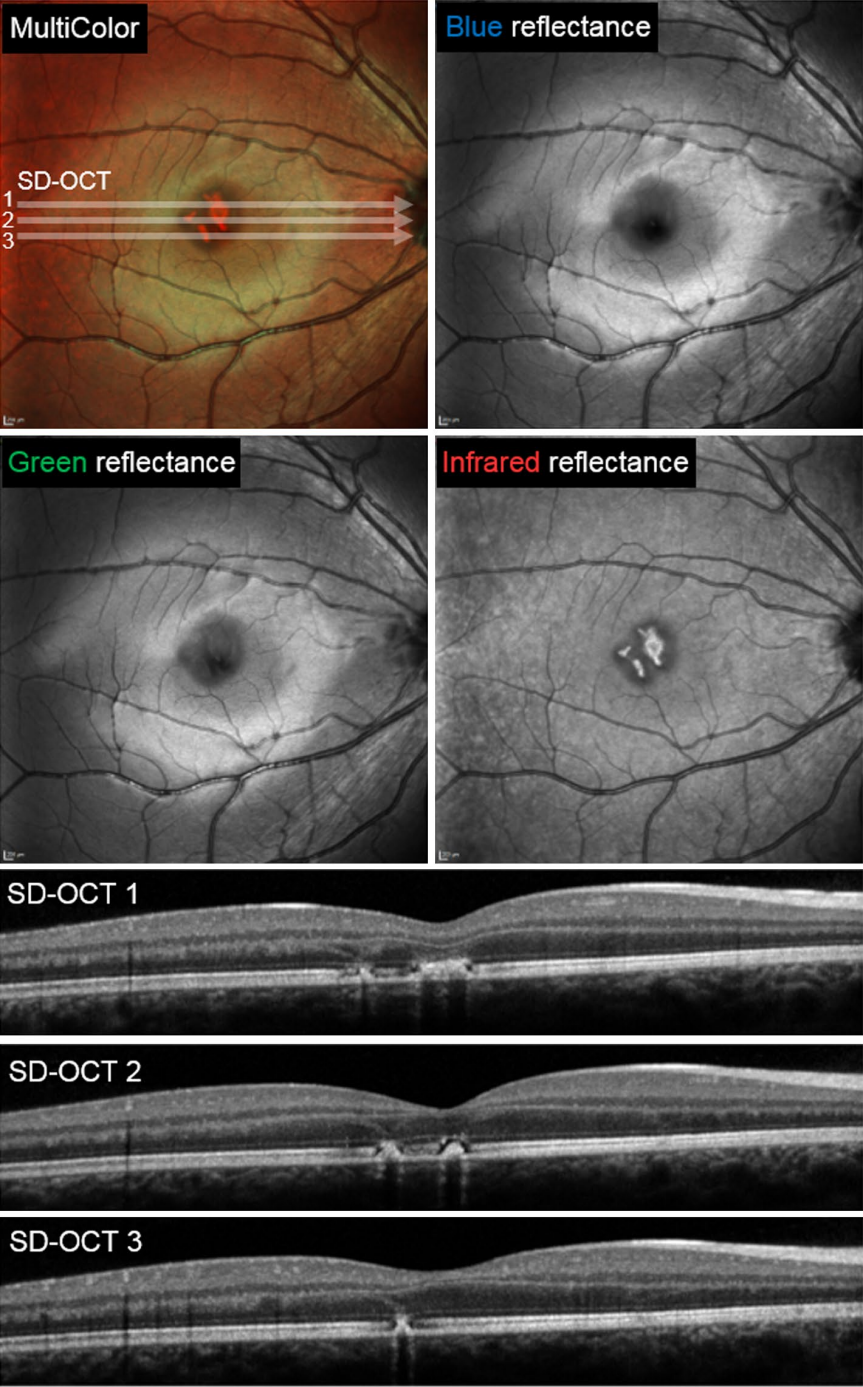
MultiColor and spectral domain optical coherence tomography findings obtained 7 days following the injury. The lesions are not seen with blue-reflectance but become increasingly apparent with the longer green and infrared reflectance wavelengths. Note that while the multicolor image sharply depicts the borders of the lesions, it may misrepresent the true color of their center as seen on funduscopy. Three SD-OCT scans through the central macula show that the vertical curvilinear hyperreflective bands are attenuated 7 days after the initial injury

**Fig. 3:**
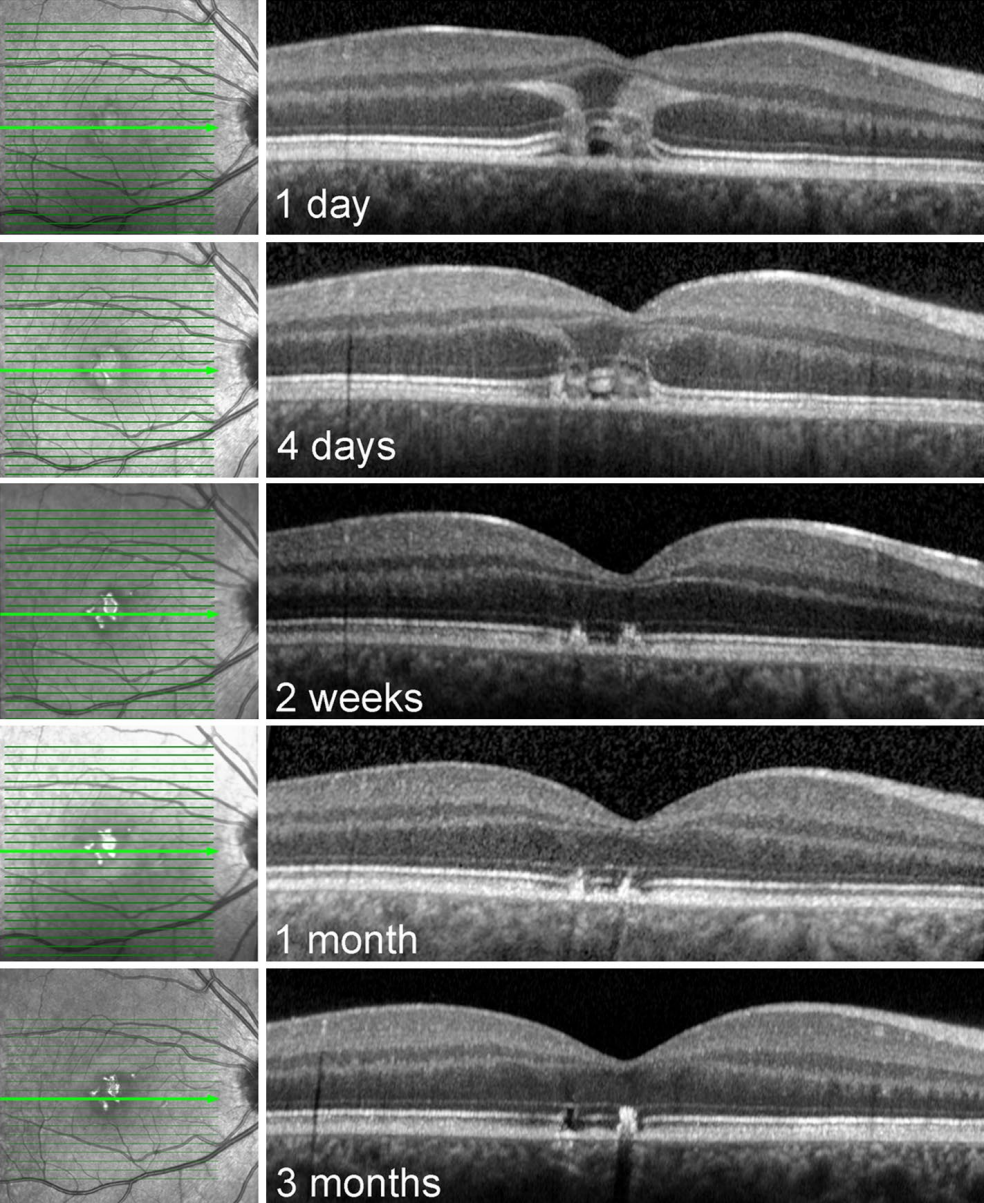
Evolution of the spectral domain optical coherence tomography: findings over 3 months. Eye-tracked, subfoveal SD-OCT line scans of the right eye are shown. The scans were taken at 1 day, 4 days, 2 weeks, 1 month and 3 months after the onset of the patient’s visual symptoms. Note how quickly the vertical curvilinear bands of hyperreflectivity resolve

**Fig. 4:**
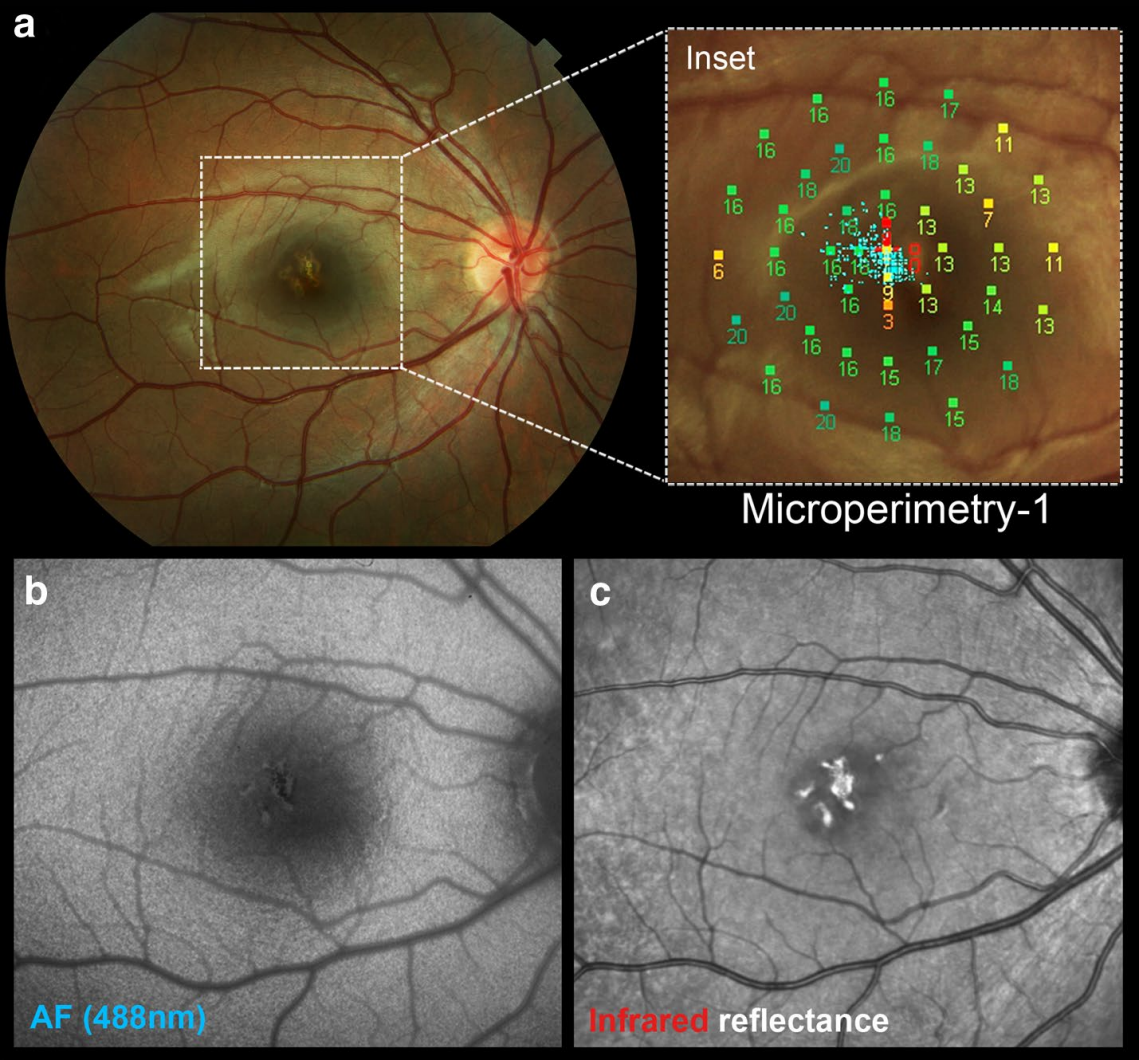
The 3 month visit findings as documented by multimodal imaging. **a** Color photograph of the right eye shows that the lesions now demonstrate central pigment hyperplasia with some surrounding depigmentation. *Inset* Microperimetry shows persistent central scotomas and slightly eccentric fixation. Visual acuity is 20/30 in the right eye. **b** Fundus (488 nm) autofluorescence shows near normalization of the acute changes and underappreciation of the lesion boundaries in comparison with the infrared image. **c** Infrared reflectance image shows high reflectivity of the macular lesions

On examination, the patient was emmetropic with best-corrected visual acuity of 20/100 in his right eye and 20/20 in his left eye. The anterior segment, lens and vitreous were unremarkable in both eyes. Funduscopic examination of the right eye revealed several sharply demarcated lesions with an excavated appearance, yellowish borders, and grey-green centers (Fig. 1a). These lesions were arranged in a vertical fashion in the central macula. The remainder of the fundus appeared normal. The funduscopic examination of the left eye showed no abnormalities. Fundus autofluorescence showed lesions that co-localized with the lesion seen in the color photographs (Fig. 1b). Fluorescein angiography performed by the referring physician 1 day following the onset of symptoms showed hyperfluorescence of the central macular lesions with minimal leakage. An additional lesion, invisible on exam, was evident nasal to the fovea (Fig. 1d). This lesion was not observed on reflectance imaging or color photography. SD-OCT performed 1 day after the injury showed curvilinear bands of dense hyperreflectivity that extended from the interdigitation layer and ellipsoid zone of the photoreceptors upwards, ending at the level of the outer plexiform layer (Fig. 1c). The hyperreflective lesions appeared to follow the Henle fibers. There were small hyporeflective cavities beneath the fovea. MultiColor and SD-OCT findings taken 7 days following the onset of symptoms are shown in Fig. 2. The evolution of the SD-OCT findings was documented over 3 months of follow-up (Fig. 3). Eye-tracked SD-OCT line scans taken at 1 day, 4 days, 2 weeks, 1 month and 3 months after injury documented the rapid evolution of the vertical curvilinear bands of hyperreflectivity. At the most recent follow-up evaluation, 3 months following the initial injury, visual acuity in the right eye had improved to 20/30. Funduscopic examination of right eye showed central pigment hyperplasia with some surrounding depigmentation of the lesions in the macula (Fig. 4a). Fundus autofluorescence showed near normalization of the acute changes (Fig. 4b), but the size of the lesions on this modality was observably smaller than the infrared reflectance image (Fig. 4c). Infrared reflectance showed high reflectivity of the macular lesions. Microperimetry showed persistent central scotomas and slightly eccentric fixation (Fig. 4d).

We were able to obtain the device that caused the injury and measure its output. While the center wavelength was 663 nm, approximating the label specification of 650 ± 10 nm, its power output was grossly mislabeled. The device had been labelled as having a maximum power output of <5 mW. However, evaluation of the device with two different optical power meters revealed values that were considerably higher than the label’s specifications. With the original batteries, the power measured 85 mW (Newport 1918-R) and 90 mW (Thorlabs PM100). With new batteries, the power measured 102.5 mW (Newport 1918-R) and 105 mW (Thorlabs PM100) (Table 1). Thus, the laser pointer was incorrectly labeled as “Class III Laser Product” (Fig. 1d) when, as per the above measurements, it should have been classified as a Class IIIB laser, which requires a key switch and a safety interlock. The parents and the patient were counseled as to the hazards of this seemingly “harmless” laser pointer device.

**Table 1:** Handheld laser pointer optical power measurements and safety reclassification

Wavelength (nm)		
Device specification	650 ± 10	
Center wavelength (FWHM)	663 (661–664)	

Optical power (mW)	Original batteries (mW)	New batteries
Device specification	<5	<5 mW
Newport 1918-R	85	102.5
Thorlabs PM100	90	105

Safety classification		Required precaution(s)
Device specification	Class III^a^	“Caution” warning label on device
Measured	Class IIIB^b^	Necessary safety components: key switch, a safety interlock dongle, a power indicator, an aperture shutter, and an emission delay

*FWHM* full width at half maximum, *nm* nanometer, *mW* microWatt

^a^Also referred to as Class IIIA (ANSI Z136.1) or 3R (IEC 60825-1)

^b^Also referred to as Class IIIB (ANSI Z136.1) or 3B (IEC 60825-1)

## Conclusions

With the increasing frequency of handheld laser induced maculopathy, its findings on multimodal imaging, particularly SD-OCT, the affected demographic, and the mid to long-term course of this injury have become more clearly defined [1, 3–8]. The coexistence of the characteristic curvilinear bands on SD-OCT along with the other findings observed in multimodal imaging is a pathognomonic pattern that, in tandem with history taking, serves to establish a prompt diagnosis. This case illustrates the temporal evolution of SD-OCT changes immediately following HLIM. It also illustrates the similarities and the discrepancies in lesion characteristics between different imaging modalities.

The recognition of the characteristic green-grey vertical lesions located in the central macula, particularly in young male patients complaining of acute to subacute vision loss, helps to establish this diagnosis. Our case demonstrates that the characteristic vertical curvilinear bands of hyperreflectivity visible in Henle’s fiber layer resolved within 2 weeks. SD-OCT changes in the course of HLIM may therefore be a short-lived finding. The importance of an immediate pattern-recognition is paramount as it may eliminate the need for unnecessary and expensive medical evaluations. More importantly, correct diagnosis may prevent further damage from the same device, at times to the fellow eye (if the injury is initially unilateral) and complications including full-thickness macular hole, choroidal neovascularization, hemorrhage, and permanent deep, central scotomata. Other reports have demonstrated evidence of persistent OCT lesions beyond the 2 week period [3], and it is therefore likely that the temporal behavior of OCT findings is related to the wavelength and power of the laser device as well as the duration of laser exposure during the initial insult.

Previous reports by our group and others [2–7] have noted that, although strict regulations by the Food and Drug Administration and the American National Standards Institute govern the safe use of medical, research and industrial lasers, there is no regulatory body monitoring the use of the widely available, easily accessible and potentially mislabeled handheld laser pointers. As evidenced by this case, the power output of these devices can greatly exceed what is listed on the label (by nearly 100 mW in our case), and the laser mislabeling allows for the omission of any mandatory safety measures for the power it actually possesses such as key switches, safety interlocks and/or the recommended use of safety goggles.

In summary, this case shows the rapid evolution of SD-OCT findings in HLIM. Awareness of these characteristic features can facilitate a prompt diagnosis of this entity even when the typical juxtafoveal vertical curvilinear opacities are absent on SD-OCT. 

## Consent

Written informed consent was obtained from the patient’s parents for publication of this case report and any accompanying images. A copy of the written consent is available for review by the Editor-in-Chief of this journal.

## Abbreviations

SD-OCT: spectral-domain optical coherence tomography
HLIM: handheld laser-induced maculopathy
